# Dynamical analysis of cellular ageing by modeling of gene regulatory network based attractor landscape

**DOI:** 10.1371/journal.pone.0197838

**Published:** 2018-06-01

**Authors:** Ket Hing Chong, Xiaomeng Zhang, Jie Zheng

**Affiliations:** 1 Biomedical Informatics Lab, School of Computer Science and Engineering, Nanyang Technological University, 639798, Singapore, Singapore; 2 Complexity Institute, Nanyang Technological University, 637723, Singapore, Singapore; Cornell University, UNITED STATES

## Abstract

Ageing is a natural phenomenon that is inherently complex and remains a mystery. Conceptual model of cellular ageing landscape was proposed for computational studies of ageing. However, there is a lack of quantitative model of cellular ageing landscape. This study aims to investigate the mechanism of cellular ageing in a theoretical model using the framework of Waddington’s epigenetic landscape. We construct an ageing gene regulatory network (GRN) consisting of the core cell cycle regulatory genes (including p53). A model parameter (activation rate) is used as a measure of the accumulation of DNA damage. Using the bifurcation diagrams to estimate the parameter values that lead to multi-stability, we obtained a conceptual model for capturing three distinct stable steady states (or attractors) corresponding to homeostasis, cell cycle arrest, and senescence or apoptosis. In addition, we applied a Monte Carlo computational method to quantify the potential landscape, which displays: I) one homeostasis attractor for low accumulation of DNA damage; II) two attractors for cell cycle arrest and senescence (or apoptosis) in response to high accumulation of DNA damage. Using the Waddington’s epigenetic landscape framework, the process of ageing can be characterized by state transitions from landscape I to II. By *in silico* perturbations, we identified the potential landscape of a perturbed network (inactivation of p53), and thereby demonstrated the emergence of a cancer attractor. The simulated dynamics of the perturbed network displays a landscape with four basins of attraction: homeostasis, cell cycle arrest, senescence (or apoptosis) and cancer. Our analysis also showed that for the same perturbed network with low DNA damage, the landscape displays only the homeostasis attractor. The mechanistic model offers theoretical insights that can facilitate discovery of potential strategies for network medicine of ageing-related diseases such as cancer.

## Introduction

Ageing is a complex process that has recently drawn much attention in the field of systems biology to elucidate the secret of longevity [1]. In a review paper [2], López-Otín et al. provided a definition of ageing: “Aging is characterized by a progressive loss of physiological integrity, leading to impaired function and increased vulnerability to death.” Moreover, López-Otín et al. highlighted 9 hallmarks of ageing and reviewed fundamental concepts about ageing [2]. These 9 hallmarks indicate the enormous complexity of the ageing process. For simplicity, this paper focuses on two of the hallmarks: genomic instability and cellular senescence. Genomic instability refers to the accumulation of DNA damage and cellular senescence is defined as permanent arrest of the cell cycle leading to an inability to proliferate [2]. These two hallmarks are chosen because they are the common and related hallmarks of ageing. However, the molecular mechanisms of cellular senescence and genomic instability have not been fully understood in a holistic and systematic way. Thus, this research aims to investigate how the regulation of senescence and genomic instability contributes to ageing using computational modeling of a gene regulatory network (GRN) in ordinary differential equations (ODEs).

Quantitative models have long been used to study the phenomenon of ageing and play important roles in understanding ageing (e.g. see the review by Edelstein-Keshet et al. [3] and the perspectives by Kirkwood et al. [4]). A complete gene network for cellular senescence is not yet available in the literature. The p53 gene network has been recognized to play important roles in senescence and ageing [5]. Therefore, we combined the core regulatory networks of p53 and cell cycle regulation to investigate the mechanism of ageing. Based on the network, we proposed a mathematical model to simulate the mechanism of ageing using dynamical systems theory. Our model can quantify the emergence of ageing in the form of potential landscape (adapted from the classic Waddington’s epigenetic landscape [6]), particularly with the notion that a cell progresses from one stable state to another stable state in the form of attractor [7]. A high level of concentration of p53 protein is a marker of cellular senescence and apoptosis [8–10], and hence we use a high level of concentration of p53 protein as an indicator of ageing. Here, the use of senescence as a marker for ageing is a simplification of the actual processes of ageing because other than senescence, stem cell exhaustion and epigenetic alteration should be markers of ageing also [2].

In this paper, we propose a mathematical model that can capture the key components of the molecular mechanisms of ageing at cellular and molecular levels and thereby shed light on the overall process of ageing. The proposed model offers a theoretical explanation of ageing by mapping the dynamics of a GRN to Waddington’s epigenetic landscape which can illustrate cell fates as attractors. The Waddington’s epigenetic landscape was originally proposed as a metaphor to describe the process of cellular differentiation as a ball rolling from the top of the hill downward towards valleys corresponding to distinct differentiated cell types [6]. A few recent papers have proposed computational methods for quantifying the Waddington’s epigenetic landscapes of development, stem cell differentiation and cancer [11–14].

Here, we propose that the Waddington’s epigenetic landscape can also be used to explain the process of ageing. To this end, we constructed a model of GRN underlying ageing which comprises 13 genes and 32 interactions. To quantify the potential landscape of the ageing network, we used a Monte Carlo computational method to calculate the potential of the landscape based on the probability distribution of state occurrences estimated by dynamical simulations from a large number (say, 100,000) of random initial conditions in the multi-dimensional state space [15]. The generated potential landscape enables us to capture the attractors as distinct cell fates or phenotypes. This method can characterize a path connecting two attractors that are formed by two unstable manifolds [15]. Two potential landscapes demonstrated three biologically interesting attractors: I) one homeostasis attractor when the accumulation of DNA damage is low; II) one attractor for the cell cycle arrest and one attractor for senescence (or apoptosis) when the accumulation of DNA damage is high. Particularly, the senescence attractor has a bigger and deeper basin of attraction than the other two attractors and it is the dominant attractor as a biological marker of ageing. These results suggest that the process of ageing can be quantified by the changes of the landscape from I to II as cells age.

Through a series of *in silico* perturbations to the network (e.g. simulating p53 inactivation), we found that the deletion of two specific interactions can lead to a change in the landscape morphology such that four attractors appear. In addition to the aforementioned three attractors, we obtained an additional attractor which corresponds to cancer, because it has low p53 protein level and high ATM level. The emergence of the cancer attractor indicates that the perturbation might enable the cells to acquire the ability to evade senescence or apoptosis. Our model analyses also suggest a link between ageing and cancer which could provide an explanation for an increase in cancer risk as cells age. Through the analyses of the potential landscapes, we make a prediction about how a cancer attractor may be eliminated from the p53 inactivated network. The model of the ageing GRN along with the proposed potential landscape method provides a framework for studying the ageing process at cellular level and uncovering the mechanisms of cellular senescence and cancer occurrence.

## Results

### A gene regulatory network model of ageing

**Fig 1:**
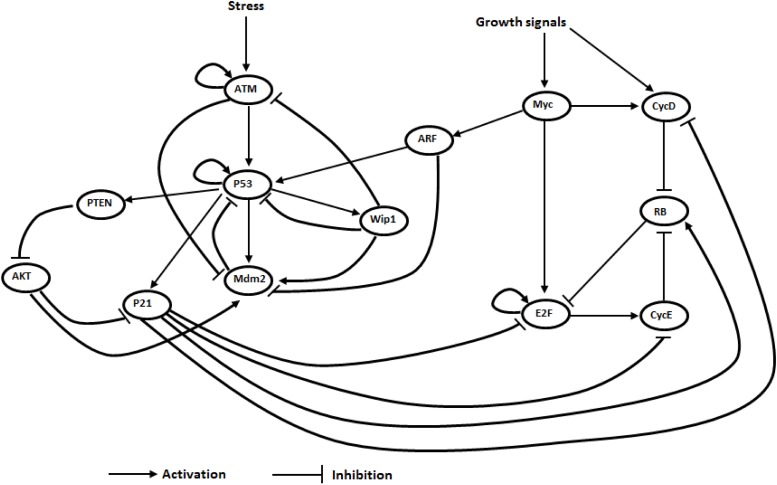
The ageing network model. The core GRN for ageing (arrows represent activations and bar arrows represent inhibitions).

To investigate the process of cellular ageing, we first propose an ageing GRN (Fig 1). The network integrates a DNA damage response network reported by Batchelor et al. [16] and a cell cycle regulation network proposed by Yao et al. [17]. We also added 4 components (ARF, PTEN, AKT, and P21) and a few interactions between the two networks (details of the model construction are given in the next subsection). The ageing GRN contains 13 genes (or proteins) as nodes and 32 directed interactions (activation or inhibition). As DNA damage response is a sophisticated network, we focus on the modeling of the core regulation of DNA damage response that contributes to ageing by selecting 13 core genes regulating DNA damage response and cell cycle: p53, Mdm2, Wip1, ATM, p21, PTEN, AKT, Myc, E2F, RB, CycE, CycD and ARF. Although, ATR is also involved in the DNA damage response, for simplicity and because DNA double-strand breaks, the most lethal form of DNA damage, are mainly detected by ATM, ATR is omitted as in other models [18–20]. There is no checkpoint protein such as 53BP1 in the GRN, because we only model the core cell cycle regulation as proposed in Yao et al (2008) [17]. There are two inputs to the model, namely the stress and the growth signals. The stress signal represents oxidative stress or DNA damaging stress signal. A mathematical model is formulated in the form of 13 Ordinary Differential Equations (ODEs), mainly with Hill functions for activation and inhibition kinetics, which are given in Methods section. The goal is to construct an ageing GRN and quantify a potential landscape with basins of attraction representing distinct cell fates (e.g. cellular senescence). High levels of p53 and p21 are used as the markers for cellular senescence [5]. A flowchart of the steps used in this study is given in Supplementary Information (S1 Fig).

### Schematic diagram of the ageing network

We constructed a mathematical model in the form of ordinary differential equations (ODEs) based on a schematic diagram (Fig 1) made by combining the p53-centered regulatory network for DNA damage response [16] and the network of cell cycle regulation from Yao et al. [17]. We integrated the two networks by adding 4 components of ARF, PTEN, AKT, and P21 in the DNA damage response, and a few interactions between DNA damage response and cell cycle regulation as follows. In the p53 regulatory network, the input is stress signal. In our model, the stress signal is denoted by Stress in Eq (4) and assumed to have a constant value of 0.3. The stress signal activates ataxia telangiectasia mutated (ATM), and it is amplified by self-activating ATM autophosphorylation [21]. We added ATM self-activation as it is important in DNA damage response. The activated ATM then activates p53, triggering responses to the stress [22]. Moreover, active ATM inhibits Mdm2 [22]. Then, p53 activates PTEN [23], Mdm2 [24], Wip1 [25], p21 [26] and itself through autoregulation [27, 28]. We also added the p53 self-activation as a positive feedback loop. Mdm2 is a key feedback regulator that inhibits p53 by promoting p53 degradation [24, 29]. As a phosphatase, Wip1 dephosphorylates ATM [30], p53 [31] and Mdm2 [32]. As a result, Wip1 inhibits ATM and p53, but activates Mdm2.

In the model, PTEN (a phosphatase) is assumed to inhibit AKT by dephosphorylating phosphatidylinositol 3,4,5-trisphosphate (PIP3) [33]. AKT activates Mdm2 [34], and inhibits p21 [35]. Meanwhile ARF inhibits Mdm2 by enhancing Mdm2 degradation [36].

In the cell cycle module from Yao et al. [17], growth signals activate Myc and Cyclin D. The strength of growth signals (denoted by GS in Eq (12)) is assumed to have a constant value of 0.2. Myc activates E2F and Cyclin D. E2F activates Cyclin E and itself through a self-activating positive feedback loop. Cyclin D and Cyclin E both inhibit RB, which inhibits E2F.

In the following we describe the interactions that are linking the 2 networks. The connection between the p53 core regulatory module and the cell cycle module lies in ARF and p21. In the cell cycle module, Myc activates ARF [37], while ARF activates p53 and inhibits Mdm2 [36]. In the p53 core regulatory module, p21 inhibits E2F [38], Cyclin D and Cyclin E [39], but p21 activates RB [40].

### Bifurcation analysis

After having formulated the model equations, our next step is to estimate the model parameters. The model contains 5 parameters: *S*, *n*, *k*, *a* and *b*. According to Li and Wang [13] *S* denotes the threshold of the sigmoidal Hill function or strength of the regulation, and *n* is the Hill coefficient which controls the steepness of the sigmoidal function. The Hill coefficient is used to represent the cooperativity in the binding process. Parameter *k* represents the spontaneous degradation rates of proteins. Based on the analyses conducted by Li and Wang [13] and some previous works [11, 41, 42], the ranges of parameter values to produce bistability are *S* ∈ [0.5,1.5] and Hill coefficient n = 4,5,…,or,8. Li and Wang chose *S* = 0.5 for the strength value, and *n* = 4 for tetramer binding [13]. Previous studies [11, 13, 41, 42] all used the self degradation rate *k* = 1. We adopted the same parameter values of *S* = 0.5, *n* = 4, and *k* = 1 as in Li and Wang [13]. By varying the value of the inhibition rate constant *b*, we attempted to find a GRN that displays three attractors corresponding to the three aforementioned distinct cell fates. Bifurcation analysis was performed (using the activation rate *a* as the bifurcation parameter) to investigate values in the parameter space for *b* that can produce multi-stability. We identified a parameter value *b* = 0.05 which corresponds to a dynamical system displaying three stable steady states (i.e. attractors).

**Fig 2:**
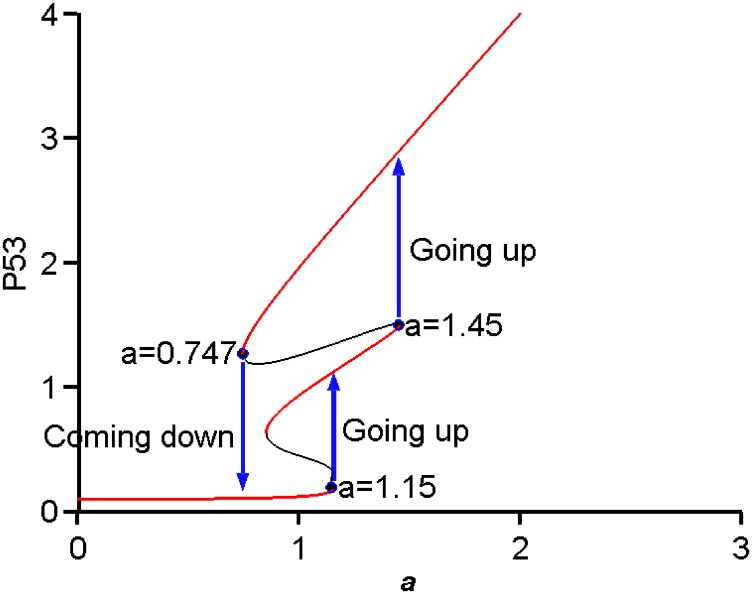
p53 bifurcation diagram. Red curves represent stable steady states and the black curves represent unstable steady states. The bifurcation diagram shows a tri-stability with hysteresis behavior. The ‘Going up’ means to turn ‘ON’ p53 and the ‘Coming down’ means to turn ‘OFF’ p53.

A bifurcation diagram of p53 against the parameter *a*, i.e. activation rate, was generated as in Fig 2. Conceptually, the activation rate represents the accumulation of DNA damage [43, 44]: a low value of *a* denotes a low accumulation of DNA damage and vice versa. The bifurcation diagram shows the existence of three stable steady states (i.e. three attractors) which could represent homeostasis (low level of p53) for low activation rate, cell cycle arrest (intermediate level of p53) for intermediate activation rate and permanent cell cycle arrest (senescence) or apoptosis (high level of p53) for high activation rate. This explanation is qualitatively consistent with the current understanding of p53 from experimental studies [5, 45, 46].

The bifurcation diagram (Fig 2) shows a tri-stability (three stable steady states) and hysteresis behavior which indicates two thresholds for activation (1.15, 1.45) to turn ‘ON’ p53 from low level (homeostasis) to intermediate level (cell cycle arrest) and then to high level of p53 (senescence or apoptosis), and one threshold (0.747) to turn ‘OFF’ p53. The bifurcation diagram also shows two ‘Going up’ jumps and one ‘Coming down’. This is a slightly different bifurcation diagram from the standard saddle-node bifurcation diagram, which typically shows only one ‘Going up’ and one ‘Coming down’. The hysteresis behavior is a special characteristic of biological switches for capturing the irreversible commitment of cell fate decision in an “all-or-none” manner, which provides a reliable mechanism for retaining the current state in the system’s memory.

**Fig 3:**
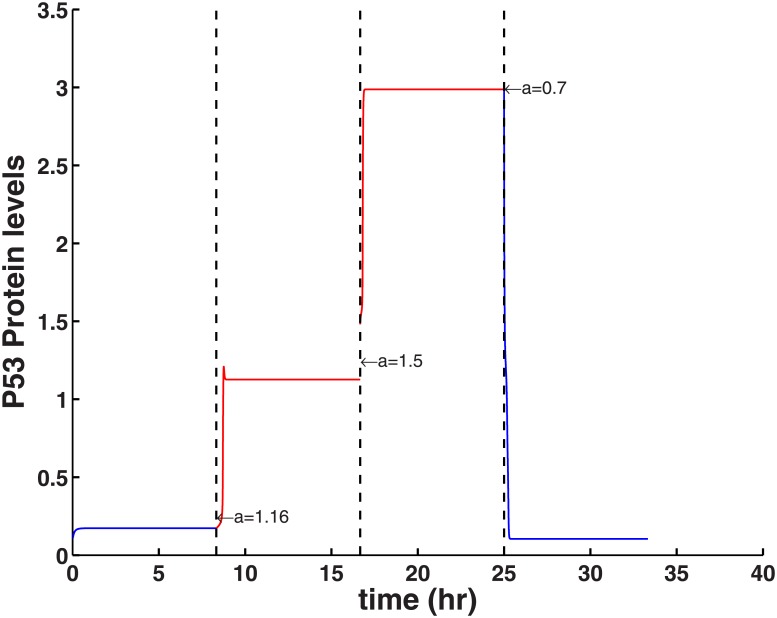
Time-course simulation of p53. The three vertical dashed lines mark two thresholds for the activation of p53 (*a* = 1.16 and *a* = 1.5) and a lower threshold to turn ‘OFF’ p53 (*a* = 0.7). The timing for the value of *a* changes is for illustration only (i.e. three different levels of p53 for three cell fates).

To illustrate the novel tri-stability behavior we simulated the changes of p53 with the value of *a* at three different time points as indicated by the vertical dashed lines (corresponding to *a* = 1.16, *a* = 1.5 and *a* = 0.7) in Fig 3. This time-course simulation illustrates the two ‘Going up’ jumps and one ‘Coming down’ as in Fig 2. The result demonstrates a molecular switch with three tipping points for controlling the three cell phenotypes of homeostasis, cell cycle arrest and senescence, represented as three distinct stable steady states. It suggests that the same GRN can produce three different phenotypes depending on the activation rate.

**Fig 4:**
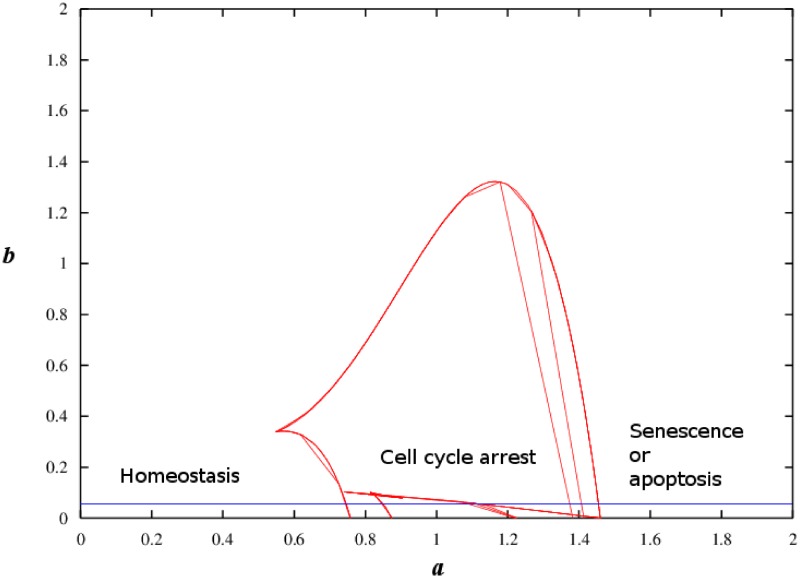
Two-parameter bifurcation diagram. Blue horizontal line indicates *b* = 0.05. Red curves represent stable steady states. This two-parameter bifurcation diagram shows that there are different combinations of values of *a* and *b* which can result in tri-stability.

A two-parameter bifurcation diagram is shown in Fig 4. It depicts the regions of the three distinct cell fates, i.e. homeostasis, cell cycle arrest and senescence (or apoptosis). For one specific case, the blue horizontal line indicates *b* = 0.05 and the region (*a* between 0.747 and 1.45) in the middle represents tri-stability. For *a* less than 0.747, the blue horizontal line falling from 0 to 0.747 represents the homeostasis state, whereas for the blue horizontal line with *a* greater than 1.45 represents the cellular senescence (or apoptosis) state. The two-parameter bifurcation diagram shows the possible cellular states in the state space of the parameters *a* and *b*.

### Recapitulation of a bistable cell cycle activation

**Fig 5:**
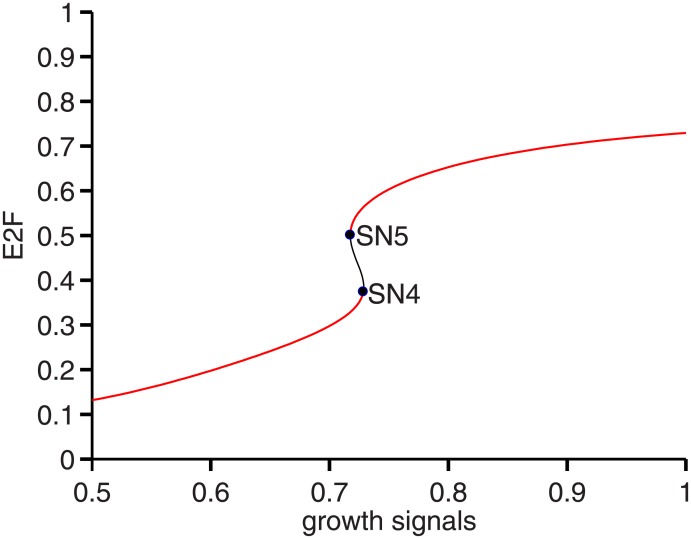
Bistable activation of E2F. Bifurcation diagram of E2F (y-axis) with respect to growth signals (x-axis).

In the work of Yao et al. (2008), it has been shown that the activation of cell cycle division is bistable with a restriction point controlled by growth signals. Next we ask if our model can capture the same property of bistability in the cell cycle activation. E2F is a key protein that plays critical role in cell cycle progression by activating a group of genes [47]. We generated a saddle-node bifurcation diagram for E2F with respect to the growth signals (shown in Fig 5). It shows that the growth signals can indeed induce E2F bistability with the hysteresis behavior. Notably, there are two threshold values of growth signals: for the upper one (SN4 with growth signals bigger than SN5) acts as the growth signal for activating cell cycle division and the lower threshold (SN5) acts to ensure an irreversible decision once the cell is committed to divide, which is consistent with the result of Yao et al. (2008).

In addition, Yao et al. (2008) have also shown that there is a monotonous increase in the steady state protein level of Cyclin D in response to increasing growth signals. Our analysis of the bifurcation diagram in S2 Fig shows that Cyclin D indeed increases monotonically with respect to growth signals. This result shows that our model can recapitulate the bistability underlying the restriction point for cell division, as observed in the experiment by Yao et al. (2008).

### Quantification of Waddington’s epigenetic landscape for ageing

The theory that ageing is a process of transition from one stable steady state representing the normal cell to another stable steady state representing the senescence (or apoptosis) has been proposed to explain the phenomena of ageing [7]. In order to demonstrate this concept, we used a novel computational method developed by ourselves (details in [15]) to quantify the Waddington’s epigenetic landscape. Similar to the self-consistent mean field method proposed by Li and Wang [13], our method defines the elevation of a state in the landscape as potential *U* = −*lnP*(*x*), where *P*(*x*) is the probability distribution of the states estimated using Monte Carlo simulations [15].

**Fig 6:**
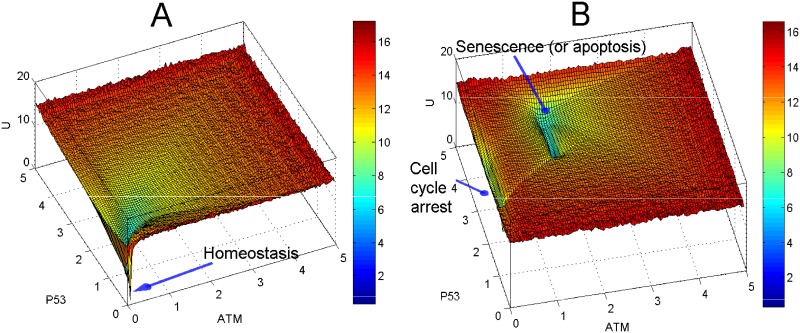
Potential landscapes for cell fate attractors (measured by p53 and ATM). (A) Landscape I: For low activation rate (*a* = 0.5, *b* = 0.05), the landscape displays one attractor (blue color) with low p53 protein corresponding to homeostasis. (B) Landscape II: For high activation rate (*a* = 1.5, *b* = 0.05), the landscape displays two attractors, one for cell cycle arrest and the other with high level of p53 protein corresponding to senescence or apoptosis. The senescence (or apoptosis) attractor is a dominant attractor of landscape II and thus a potential biomarker of ageing.

To display the 3D view of the potential landscape, we selected p53 and ATM as the two dimensions of the state space, because high p53 protein level can activate senescence and apoptosis [8–10] and ATM is a key component for detecting stress signals [48]. The third dimension is defined as the potential *U*. The potential landscapes (Fig 6(A) and 6(B)) illustrate the cell fate attractors (with local minimum potential or local maximum probability) for different values of activation rate. For cells with low activation rate corresponding to low DNA damage, the landscape displays one attractor with low levels of p53 and ATM, which correspond to cell homeostasis (Fig 6(A)) and it is named landscape I. For ageing cells with a high activation rate which corresponds to high accumulation of DNA damage causes the morphology of the landscape to change. The landscape displays two attractors which represent cell cycle arrest (with intermediate p53 level) and senescence (or apoptosis) indicated by high levels of p53 and ATM. Ageing cells tend toward the dominant attractor (i.e. bigger basin of attraction) of senescence or apoptosis (Fig 6(B)) in the new landscape and let us name it landscape II. Similar results of the potential landscape were quantified when p21 and ATM were used as coordinates of the state space (see S3 Fig), where high p21 is used as a marker of cellular senescence [5]. The process of ageing can be described by the change from landscape I to landscape II. This shift from homeostasis to senescence (or apoptosis) could explain why ageing occurs as a result of accumulated DNA damage represented by the increased value of *a*. The resulting accumulation of senescent cells are often observed in aged human tissues such as skin, liver and lung [49].

Parameter sensitivity analyses of *a* and *b* were performed. The results of the potential landscape show that the landscape shape is robust to changes in the value of *b*, as all the potential landscapes display qualitatively consistent morphologies for different values of *b* (see S1 Table). For a low value of *a* = 0.5 (first row in S1 Table) and *b* in the range from 0.05 to 0.8, the landscape displays one basin of attraction (with a low p53 level which indicates homeostasis and an intermediate p53 level which indicates cell cycle arrest). However, for a high value of *a* (*a* from 1 to 1.4, rows 3-4 in S1 Table), a potential landscape shows two basins of attraction, which correspond to cell cycle arrest (intermediate level of p53) and senescence or apoptosis (with a high p53 level).

**Fig 7:**
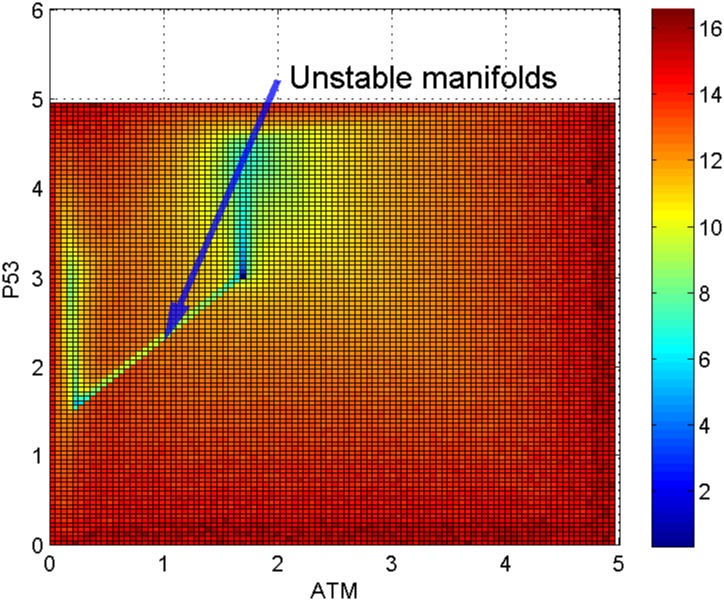
Top view of landscape II in Fig 6(B). There is a line connecting the two attractors. The line is formed by two unstable manifolds representing a kinetic path between the cell cycle arrest and senescence (or apoptosis) basins of attraction.

Looking at Fig 7 which is a top view of the landscape in Fig 6(B), we can observe a path (or valley) between the cell cycle arrest attractor and the senescence (or apoptosis) attractor. Similar to an example of a well-studied synthetic toggle switch (as in [15]), the path connecting the two attractors is formed by two unstable manifolds of a saddle point. The saddle point marks a barrier height for separating the two attractors [15]. Note that Li and Wang have proposed the potential differences between a saddle point and the two attractors as the barrier heights in the potential landscape [11–13]. Figs 6(B) and 7 suggest an interpretation that there is a path (represented by the unstable manifolds) connecting the two attractors corresponding to cell cycle arrest and senescence (or apoptosis) respectively.

### Link between ageing and cancer attractors

The aforementioned landscapes for cell fate decisions in Fig 6 demonstrate three types of attractors: 1) the homeostasis attractor when both p53 and ATM are low; 2) the cell cycle arrest attractor when p53 is intermediate and ATM is low; 3) the senescence or apoptosis attractor when both p53 and ATM are high. There are other combinations of the protein levels with potential biological significance, such as low level of p53 which indicates cancer.

**Fig 8:**
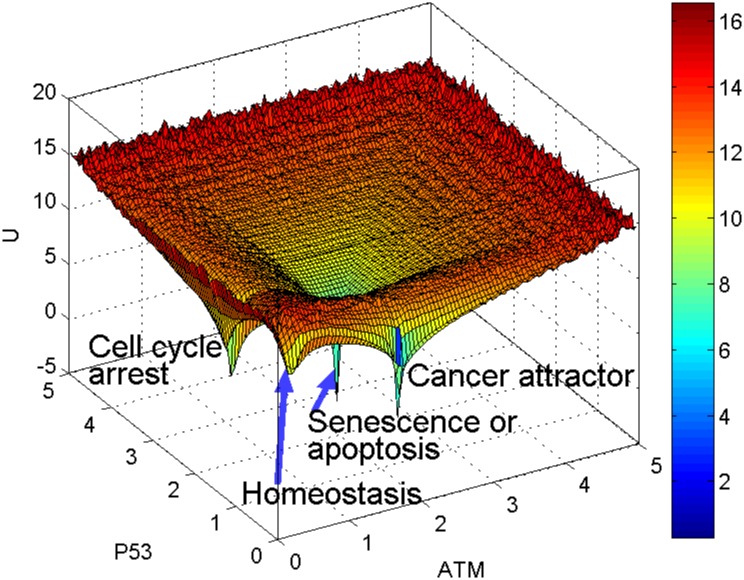
Emergence of cancer attractor. For the inactivation of p53 with the deletion of two interactions with p53 (the activation of p53 by ATM and by ARF respectively), the landscape displays an additional attractor with low p53 level and high ATM (*a* = 1.5, *b* = 0.05). This attractor represents cancer because low p53 level leads to p53 inactivation that enables cells to acquire the ability to evade cellular senescence or apoptosis.

**Fig 9:**
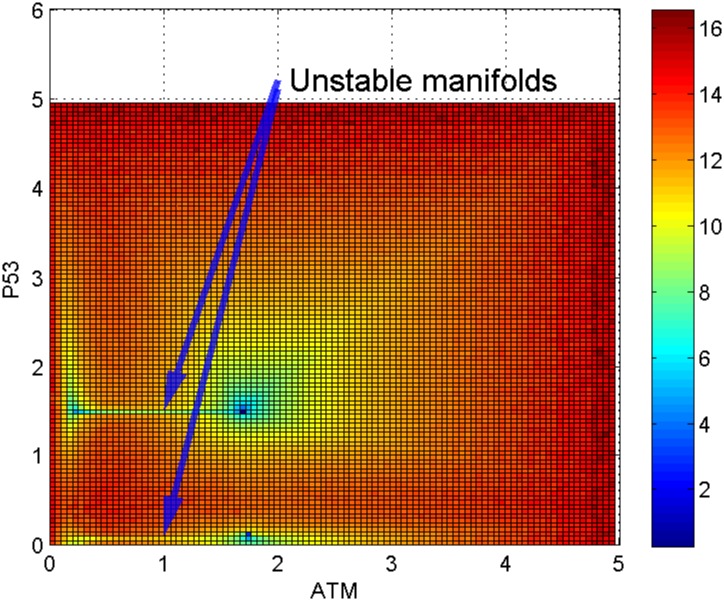
Connection between ageing and cancer attractors. Top view of Fig 8 shows: 1) there is a line (represents two unstable manifolds) connecting cell cycle arrest and senescence (or apoptosis) attractors; 2) another line connecting homeostasis and cancer attractors.

Next we perform *in silico* perturbations to the network to study the link between ageing and cancer through p53. Inactivation of p53 has been reported in about half of known cancers [50]. To mimic the p53 inactivation, we deleted a few combinations of interactions with p53 and obtained the potential landscapes (see S2 Table). One interesting perturbation is the deletion of two interactions with p53 (i.e. the activation of p53 by ATM and by ARF respectively), which produces four attractors in the potential landscape. The effects of the perturbations to the network were captured by the emergence of a new attractor in addition to the three aforementioned attractors (in the landscape region with low p53 and high ATM) (Figs 8 and 9). We named this attractor as “cancer attractor” because of low p53 protein level and high ATM. Low p53 protein level represents the absence of p53 tumor suppression function. The global view of the four basins of attraction cannot be obtained if we only look at the bifurcation diagram, e.g. S4 Fig. only shows two stable steady states for p53. More information about the cancer attractor with different values of the parameters *a* and *b* can be found in S3 Table.

This *in silico* prediction suggests that the p53 inactivation through the deletion of two interactions with p53, namely the activation by ATM and the activation by ARF, may cause an irreversible cell fate probably corresponding to cancer. This prediction is consistent with the experimental observation reported in the literature that the inactivation of p53 causes cancer [46, 50]. The landscape also suggests that the ageing network with high activation rate (*a* = 1.5) along with p53 inactivation (low p53 protein level) can model the emergence of cancer. The analysis of the landscape with p53 inactivated reveals a potential link between ageing and cancer.

To study the link between the two attractors, we examine the shape of landscape region nearby. From Fig 9, the top view of Fig 8, we identified two lines connecting the two pairs of attractors. As mentioned earlier, these lines indicate unstable manifolds composed of saddle points. The first path (pointed by the arrow at the top in Fig 9) indicates the link between cell cycle arrest and senescence (or apoptosis) basins of attraction as discussed earlier. The second path at the bottom is between the homeostasis attractor and the cancer attractor, which suggests the possibility of a normal cell being transformed into a cancer cell. The depth of an attractor is represented by dark blue in the color bar corresponds to the probability of state occurrence. The cancer attractor looks deeper (with darker blue color) than the homeostasis attractor in Fig 9, of which the biological meaning is still unclear and needs further analysis and interpretation.

**Fig 10:**
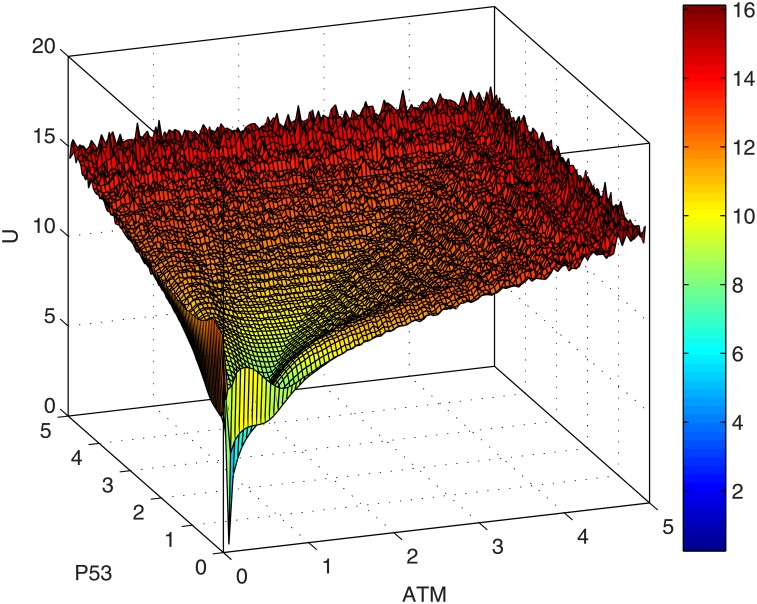
No cancer attractor for low accumulation of DNA damage. In response to the inactivation of p53 with the deletion of two interactions to p53 (the activation of p53 by ATM and by ARF respectively), the landscape displays only one attractor for homeostasis and no cancer attractor for *a* = 0.5 and *b* = 0.05, which might correspond to young cells with low accumulation of DNA damage.

An important question in Biomedicine is why the risk of cancer tends to increase with ageing. Here we use our model to provide a theoretical answer to this question. For the same perturbation deleting two interactions (activation of p53 by ATM and by ARF), we performed sensitivity analyses for the parameters *a* and *b*. Based on the results of the landscape analyses (S3 Table), we found that cancer risk increases for ageing cell when the potential landscape shows a cancer attractor. Ageing cell with high accumulated DNA damage corresponds to a higher value of *a* in our model. For example, when *a* = 1 (S3 Table) and *a* = 1.5 (Figs 8 and 9) we found the emergence of cancer attractor. By contrast, when *a* = 0.5 which corresponds to young cells with low level of DNA damage, we found no cancer attractor. Rather, the landscape shows only one attractor of homeostasis (Fig 10). This result suggests that cells with low accumulation of DNA damage are less likely to become cancer cells.

**Fig 11:**
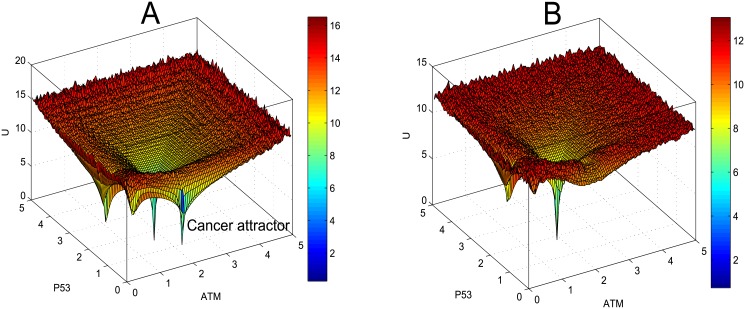
Disappearance of cancer attractor. (A) The landscape with the cancer attractor (*a* = 1.5, *b* = 0.05). (B) For *a* = 1.5, the cancer attractor disappears when the inhibition rate *b* is increased to 1.2.

Next we investigate how, by perturbing the network interactions, the existence of the cancer attractor may be eliminated. In Fig 8, we observed the emergence of the cancer attractor when p53 is inactivated by deleting two interactions. Based on the parameter sensitivity analyses (S3 Table), we found that by increasing the inhibition rate *b* the cancer attractor may be eliminated. The landscapes in Fig 11 illustrate that the cancer attractor can be eliminated when the inhibition rate *b* is increased to 1.2 (Fig 11(B)). This theoretical observation could be an interesting hint for cancer medicine. However, we also observe that the increase of *b* leads to a deeper attractor of senescence or apoptosis relative to other attractors. More detailed analysis would be needed to interpret the biological meaning of this change of landscape when the values of both parameters *a* and *b* are high.

## Discussion

The Waddington’s epigenetic landscape is a classic metaphor proposed by Waddington in 1957 for explaining the processes of embryonic development and cell differentiation [6]. The main idea is that valleys (or attractors) in a landscape correspond to the stable differentiated cells and the process of cell differentiation is like the rolling of a ball from the top of the mountain to one of the valleys. Although one conceptual landscape model for ageing with senescence and apoptosis attractors has been proposed by Kriete and Cloutier [7], a quantitative model has not yet been constructed to model the ageing process. Ageing is known to be highly complex [1]. It is reasonable to model ageing GRN using the framework of Waddington’s epigenetic landscape and study the mechanism of cellular ageing by mapping from genotype to phenotype with the landscape model.

Mathematical and computational methods that quantify quasi-potential energy for modeling the Waddington’s epigenetic landscape based on GRN models have been developed over the years [51]. The quantitative landscape models can provide important information of multi-stability in the form of basins of attraction, which is not possible with the local stability analysis of bifurcation diagram in conventional dynamical systems studies [52]. Thus, the potential landscape method which quantifies the basins of attraction is crucial for understanding the cellular dynamics driven by GRNs. Besides differentiation and ageing, cancer is another cellular phenotype modeled by the attractor landscape. The hypothesis of cancer attractor has been proposed since the 1970s [53–57]. According to Huang et al. (2009), attractors represent stable states of gene expression patterns corresponding to cell types, and some of the abnormal attractors (or cell types) are called cancer attractors [54]. Recently, the cancer attractors in the Waddington’s epigenetic landscape have been quantified in two theoretical models proposed by Li and Wang [12, 13]. They constructed a 32-gene cancer network [12] and a 6-gene core cancer stem cell GRN [13], and then used their self-consistent mean field approximation method to quantify the global potential of the GRNs [12, 13].

In this study, we proposed a 13-gene ageing GRN and uncovered the global potential landscapes for cellular ageing by using our Monte Carlo method to quantify the probability distribution of non-equilibrium and equilibrium states [15]. To the best of our knowledge this is the first quantitative study that uses the metaphor of Waddington’s epigenetic landscape to model cellular ageing. For different levels of activation rate representing different levels of accumulated DNA damage, there are changes in the number of attractors in the landscape. In our model of ageing landscape, we demonstrated that the landscape of the young cells with low accumulation of DNA damage shows one homeostasis attractor, whereas high accumulation of DNA damage causes the landscape to display an ageing attractor (i.e. cellular senescence and apoptosis attractor). In the ageing state (i.e. the senescence attractor), cells lose the ability to proliferate and as such cannot replenish enough new cells to sustain the functions of a tissue.

The attractors were quantified using our Monte Carlo method [15]. We have performed a few case studies on GRNs and the results show that our Monte Carlo method can quantify Waddington’s epigenetic landscape with more details comparable to the self-consistent mean field approximation method [13]. In addition, the Monte Carlo method can capture kinetic paths between two attractors, saddle point and the intermediate details of the Waddington’s epigenetic landscape [15]. To investigate the dynamics of cellular ageing more quantitatively, we have also conducted the time-course simulations with random initial conditions. By assuming the self degradation rate as *k* = 1 hour^−1^ and other rate constants time unit in hour, our simulation predicted that when the protein levels converge to a stable steady state of high p53 protein, cells can change to the senescent state within 20 hours (S5 Fig).

Our *in silico* experiments mimicked the perturbations to the GRN by deleting two interactions with p53 in the network. The notion of mutation in terms of the network rewiring (i.e. deletion or addition of edges or nodes in the GRN) was proposed by Huang et al. (2009) who reported that the mutations can lead to “a change in the landscape topography” [54]. Our *in silico* perturbation experiments demonstrated this notion by showing that the inactivation of p53 by deleting two edges leads to the emergence of a cancer attractor. Driven by the perturbed network, the simulated cells acquire the capability to evade the cellular senescence or apoptosis, which reveals the link between ageing and cancer. In addition, we made a prediction that increasing the parameter value *b*, the inhibition rate constant, could eliminate the cancer attractor from the potential landscape. This prediction proposed a possible strategy for cancer treatment. Perturbation to the network interactions for drug discovery is a strategy of network medicine proposed by Barabási et al. [58] and Creixell et al. [56].

One of the benefits of cellular senescence is the ability to prevent the effects of DNA damage and mutations in a cell from propagating to daughter cells. Cellular senescence contributes to the suppression of tumorigenesis, and a high p53 protein level can be used as a marker for cellular senescence as reported in recent experiments [9]. However, a side effect of tumor suppression by cellular senescence is contribution to ageing. It is suggested that the GRNs are highly optimized through evolution to strike a balance for cellular fitness. Nelson and Masel (2017) recently constructed a mathematical model of intercellular competition in genetic evolution, which also suggests that one either chooses the accumulation of senescent cells or faces the cancer risk, and therefore cellular ageing is unavoidable [59].

One limitation of our model is the use of a common degradation rate, *k* = 1, for all the proteins. As an attempt to overcome this limitation, we have tried using more realistic and individual protein degradation rates estimated from experiments as reported in the literature. From the half-lives of proteins we can use the formula $k=\frac{ln(2)}{half-life}$ to obtain a list of different degradation rates (Table A in S1 Text). Using such protein degradation rates, we quantified and plotted the Waddington’s epigenetic landscapes. Unfortunately, the landscapes each display only a single stable attractor for both *a* = 0.5 and *a* = 1.5 (Fig A and Fig B in S1 Text). Bifurcation analyses were performed to find possible parameter values of *a* or *b* that correspond to multi-stability. However, the bifurcation analyses (Fig C and Fig D in S1 Text) have also shown that using the new set of protein degradation rates it is not possible to produce multi-stability. Although the degradation rate *k* = 1 used in our model is of the same order of magnitude as the range of parameters between 0.06 and 1.40 determined using experimental data, the dynamic behaviors of the model are different when using these experimentally estimated parameter values. One possible reason is that the estimated values may not be accurate enough. Another reason could be that the computational model of the ageing GRN is focused on the core regulatory circuit of DNA damage response and cell cycle control and therefore is not comprehensive enough. In the future some crucial molecular interactions should be identified and added to the model. Moreover, an important future direction is to utilize statistical learning methods to infer network structure and estimate parameter values from real data.

In conclusion, we demonstrated that the molecular interactions in a GRN can control the cellular dynamics of ageing. The accumulated DNA damage may affect the phenotypes of ageing in the form of emergence or disappearance of attractors in the potential landscape [52]. Our *in silico* perturbations to the network predicted that p53 inactivation could lead to the emergence of a cancer attractor. This cancer attractor suggests how cells can evade the cell fate of senescence or apoptosis. Although the proposed model is based on a core ageing GRN, it gives insights into the mechanisms of ageing and cancer, and thereby offers a theoretical framework for further analysis of ageing. However, besides the above-mentioned use of common parameters, the conceptual model proposed here has a few limitations. First, the process of ageing characterized by the genomic instability might have been oversimplified in the form of accumulated DNA damage. Here, we assumed that the GRN is the same for different cells, i.e. “hard-wired” in the genome [52], although the genome instability due to accumulation of DNA damage can cause changes in the gene expression. Secondly, the ageing GRN contains only the core networks of p53 and cell cycle regulation. In the future work, the ageing network model should be expanded to a more comprehensive and detailed system of DNA damage response and cell cycle regulation. It is also desirable to incorporate other hallmarks of ageing such as epigenetic modifications.

## Materials and methods

### Model equations

The molecular interactions in the network described in the previous subsection can be written into ODEs. The Hill function with coefficient (*n*) set to 4 is used to model the activation and inhibition kinetics, and the kinetics of degradation is modeled with a linear component in the ODEs as in [13, 41]. For example, the ODE for the rate of change of variable *X* that is subject to one activation and one inhibition is formulated as

$$\begin{array}{c} \frac{dX}{dt}=a\frac{X_{a}^{n}}{S^{n}+X_{a}^{n}}+b\frac{S^{n}}{S^{n}+X_{b}^{n}}-k\cdot X, \end{array}$$

where the first term on the right hand side denotes the activation of *X* by *X*_a_, the second term denotes the inhibition of *X* by *X*_*b*_) and the third term represents the linear degradation of *X*. Our ODEs (modeling dynamics of 13 molecular species representing proteins) are as follows:

$$\begin{array}{ccc} \frac{dP53}{dt} & =a\frac{ARF^{n}}{S^{n}+ARF^{n}}+a\frac{P53^{n}}{S^{n}+P53^{n}}+a\frac{ATM^{n}}{S^{n}+ATM^{n}} & \\ & +b\frac{S^{n}}{S^{n}+Mdm2^{n}}+b\frac{S^{n}}{S^{n}+Wip1^{n}}-k\cdot P53 \end{array}$$ (1)

$$\begin{array}{ccc} \frac{dMdm2}{dt} & =a\frac{P53^{n}}{S^{n}+P53^{n}}+a\frac{AKT^{n}}{S^{n}+AKT^{n}}+a\frac{Wip1^{n}}{S^{n}+Wip1^{n}} & \\ & +b\frac{S^{n}}{S^{n}+ATM^{n}}+b\frac{S^{n}}{S^{n}+ARF^{n}}-k\cdot Mdm2 \end{array}$$ (2)

$$\begin{array}{cc} \frac{dWip1}{dt} & =a\frac{P53^{n}}{S^{n}+P53^{n}}-k\cdot Wip1 \end{array}$$ (3)

$$\begin{array}{cc} \frac{dATM}{dt} & =a\frac{ATM^{n}}{S^{n}+ATM^{n}}+a\frac{Stress^{n}}{S^{n}+Stress^{n}}+b\frac{S^{n}}{S^{n}+Wip1^{n}}-k\cdot ATM \end{array}$$ (4)

$$\begin{array}{cc} \frac{dP21}{dt} & =a\frac{P53^{n}}{S^{n}+P53^{n}}+b\frac{S^{n}}{S^{n}+AKT^{n}}-k\cdot P21 \end{array}$$ (5)

$$\begin{array}{cc} \frac{dPTEN}{dt} & =a\frac{P53^{n}}{S^{n}+P53^{n}}-k\cdot PTEN \end{array}$$ (6)

$$\begin{array}{cc} \frac{dAKT}{dt} & =b\frac{S^{n}}{S^{n}+PTEN^{n}}-k\cdot AKT \end{array}$$ (7)

$$\begin{array}{cc} \frac{dMyc}{dt} & =a\frac{GS^{n}}{S^{n}+GS^{n}}-k\cdot Myc \end{array}$$ (8)

$$\begin{array}{ccc} \frac{dE2F}{dt} & =b\frac{S^{n}}{S^{n}+P21^{n}}+a\frac{E2F^{n}}{S^{n}+E2F^{n}}+a\frac{Myc^{n}}{S^{n}+Myc^{n}} & \\ & +b\frac{S^{n}}{S^{n}+RB^{n}}-k\cdot E2F \end{array}$$ (9)

$$\begin{array}{cc} \frac{dRB}{dt} & =a\frac{P21^{n}}{S^{n}+P21^{n}}+b\frac{S^{n}}{S^{n}+CycD^{n}}+b\frac{S^{n}}{S^{n}+CycE^{n}}-k\cdot RB \end{array}$$ (10)

$$\begin{array}{cc} \frac{dCycE}{dt} & =a\frac{E2F^{n}}{S^{n}+E2F^{n}}+b\frac{S^{n}}{S^{n}+P21^{n}}-k\cdot CycE \end{array}$$ (11)

$$\begin{array}{cc} \frac{dCycD}{dt} & =a\frac{Myc^{n}}{S^{n}+Myc^{n}}+a\frac{GS^{n}}{S^{n}+GS^{n}}+b\frac{S^{n}}{S^{n}+P21^{n}}-k\cdot CycD \end{array}$$ (12)

$$\begin{array}{cc} \frac{dARF}{dt} & =a\frac{Myc^{n}}{S^{n}+Myc^{n}}-k\cdot ARF \end{array}$$ (13)

The default parameter values that we chose here are: *S* = 0.5, *n* = 4, *k* = 1, *a* = 1 and *b* = 0.05. Parameters *a* and *b* can have different values for individual figures plotted by simulating different conditions of cells.

### Dynamic simulations

Time-course simulations were carried out using XPPAUT and bifurcation analyses were done using XPP-AUTO [60] and Osclli8 [61]. The XPPAUT code is given in S1 Code. Potential landscape quantifications were performed using a Monte Carlo computational method in MATLAB [15]. The MATLAB code for drawing Waddington’s epigenetic landscape is given in S2 Code.

### Computational method for quantifying potential landscape

We used a Monte Carlo computational method for quantifying potential landscape of a GRN formulated in the form of ODEs. This computational method is described in another manuscript we have submitted earlier [15]. The Monte Carlo method is based on a large number of time-course simulations with random initial conditions. Each of the time-course trajectories is projected to a 2-dimensional plane that is divided into grid boxes to estimate the probability distribution *P*(*x*), which represents the probability distribution of non-equilibrium and equilibrium states. Then a quasi-potential *U* = −*lnP*(*x*) can be calculated. Based on the quasi-potential *U*, a potential landscape can be plotted.

## Supporting information

S1 Fig Flowchart of the study. A flowchart of this study from the ageing GRN construction to model analysis and interpretation.
(PDF)

S2 Fig Monotonously increasing steady state for Cyclin D. Bifurcation diagram of Cyclin D (*y*-axis) with respect to growth signal (*x*-axis).
(PDF)

S3 Fig Potential landscape for cell fate attractors (p21 and ATM). (A) Landscape I: For a low activation rate (*a* = 0.5), the landscape displays one attractor with low p21 protein concentration corresponding to homeostasis. (B) Landscape II: For a high activation rate (*a* = 1.5), the landscape displays two attractors, one for cell cycle arrest and the other with high p21 protein concentration corresponding to senescence or apoptosis.
(PDF)

S4 Fig Bifurcation diagram of p53 (after deleting two interactions with p53). Red lines represent stable steady states.
(PDF)

S5 Fig Time-course simulations to determine how quickly cells can change their state to senescent state. Time-course simulations for *a* = 1.5, *b* = 0.05, *k* = 1, *S* = 0.5 and *n* = 4 with random initial conditions which represent different cell states show that proteins converge to the stable steady state with a high p53 protein level indicating senescent state within about 20 hours.
(PDF)

S1 Table Potential landscapes for parameter sensitivity analysis. Parameter sensitivity analyses were performed. For different combination of parameter values of *a* and *b*, the landscapes in 3-dimensional views are shown in the table.
(PDF)

S2 Table *In silico* perturbations of p53 inactivation and the corresponding potential landscape (top view and 3D view). *In silico* network perturbations were performed with *a* = 1.5 and *b* = 0.05.
(PDF)

S3 Table Potential landscapes from parameter sensitivity analysis for mimicking p53 inactivation (3D view). Parameter sensitivity analyses were performed for the perturbed network. Potential landscapes in 3-dimensional views are shown in the table.
(PDF)

S1 Text Supplementary information about the attempt to use more realistic individual protein degradation rates. (PDF)

S1 Code The XPPAUT source code to draw bifurcation diagram. (ODE)

S2 Code The MATLAB source code to draw Waddington’s epigenetic landscapes. (ZIP)
