# Supplementary figures and images for: Dynamical analysis of cellular ageing by modeling of gene regulatory network based attractor landscape
Source: PLoS One. 2018 Jun 1;13(6):e0197838. doi: 10.1371/journal.pone.0197838 (PMC5983441; doi:10.1371/journal.pone.0197838)

S1 Fig.

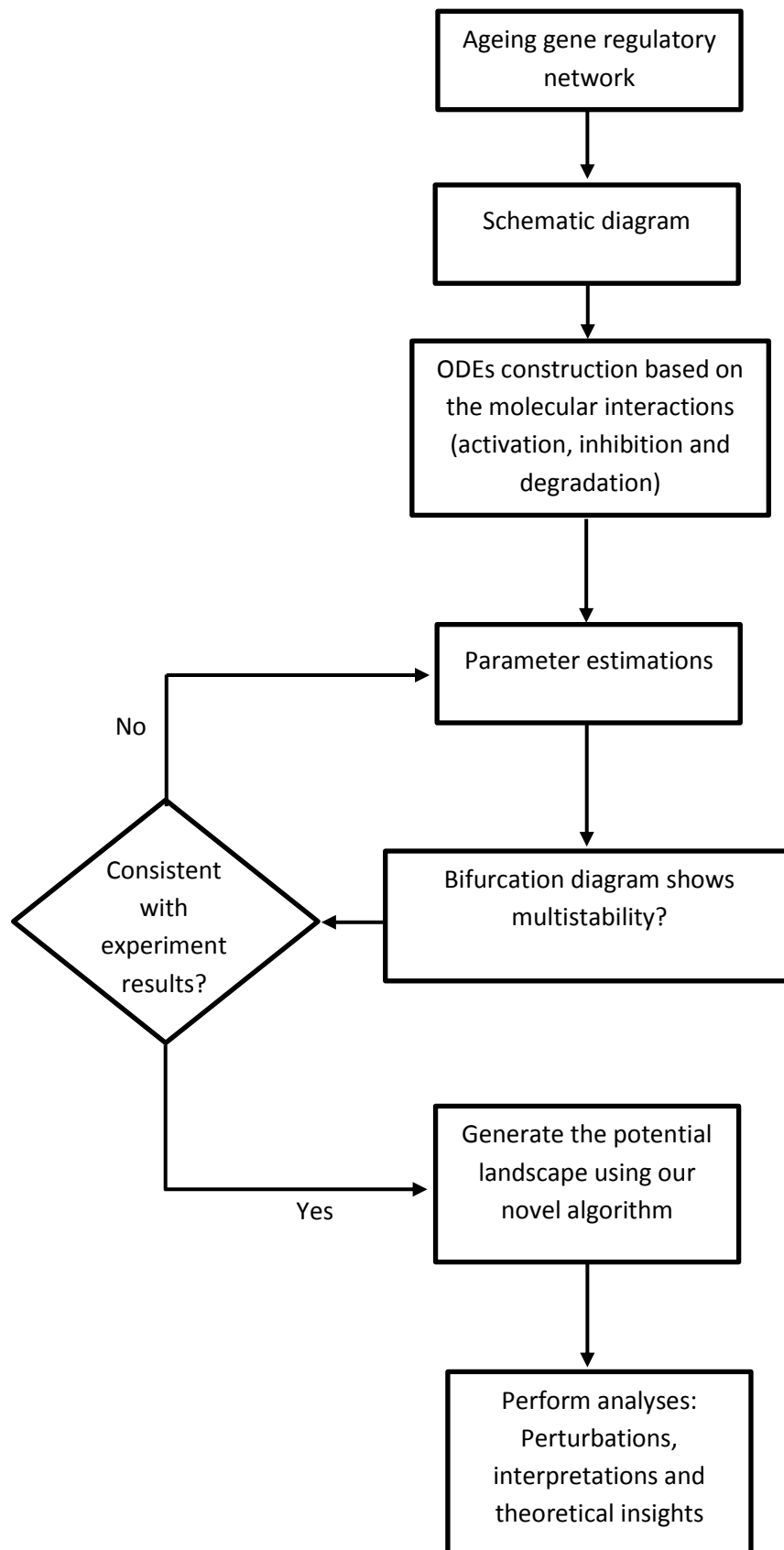

Supplement: S1 Fig — A flowchart of this study from the ageing GRN construction to model analysis and interpretation. (PDF) [file pone.0197838.s001.pdf]

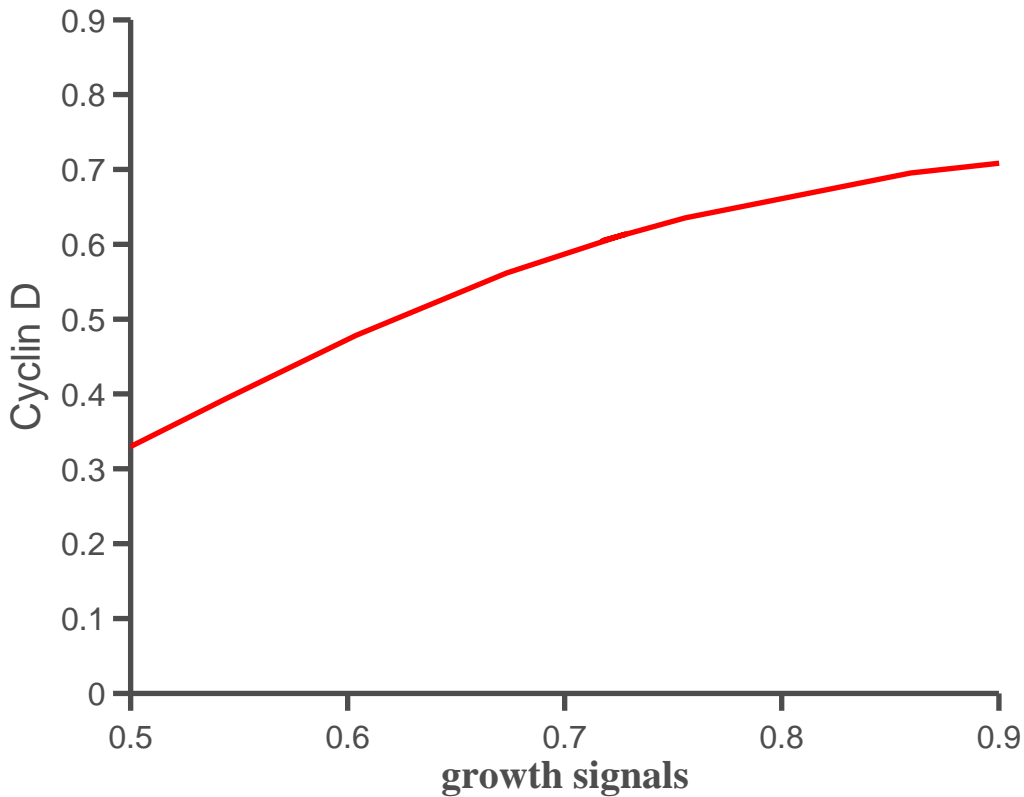

Supplement: S2 Fig — Bifurcation diagram of Cyclin D (y-axis) with respect to growth signal (x-axis). (PDF) [file pone.0197838.s002.pdf]

A

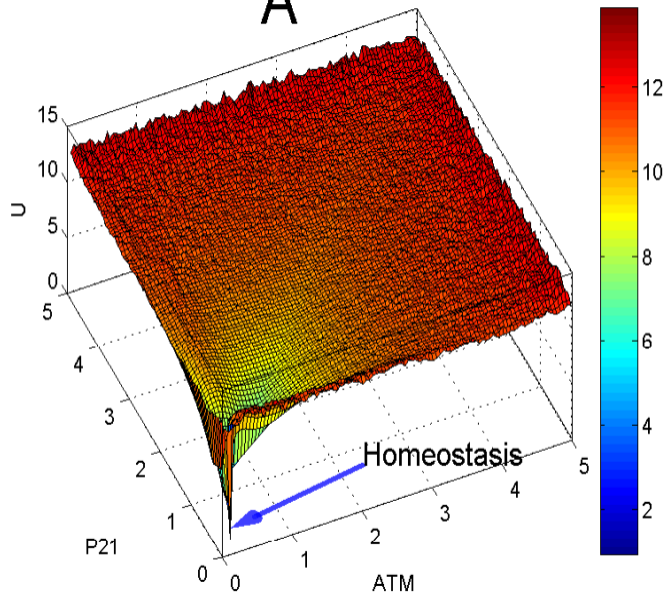

B

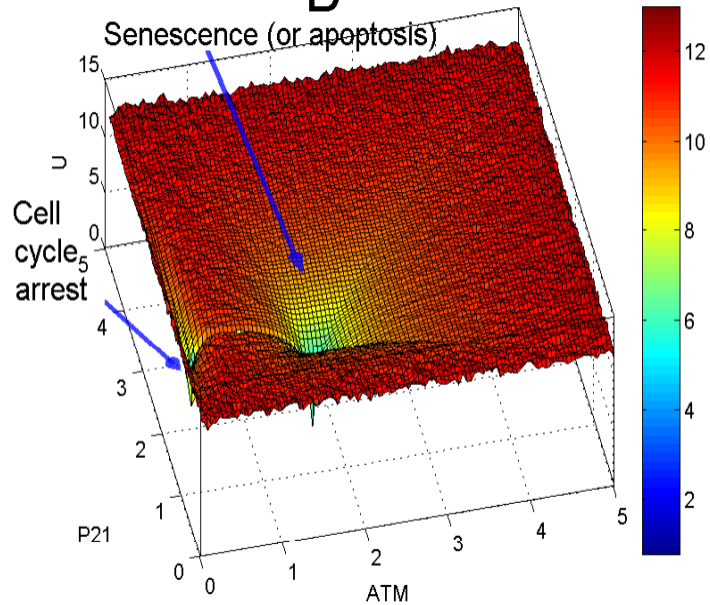

Supplement: S3 Fig — (A) Landscape I: For a low activation rate (a = 0.5), the landscape displays one attractor with low p21 protein concentration corresponding to homeostasis. (B) Landscape II: For a high activation rate (a = 1.5), the landscape displays two attractors, one for cell cycle arrest and the other with high p21 protein concentration corresponding to senescence or apoptosis. (PDF) [file pone.0197838.s003.pdf]

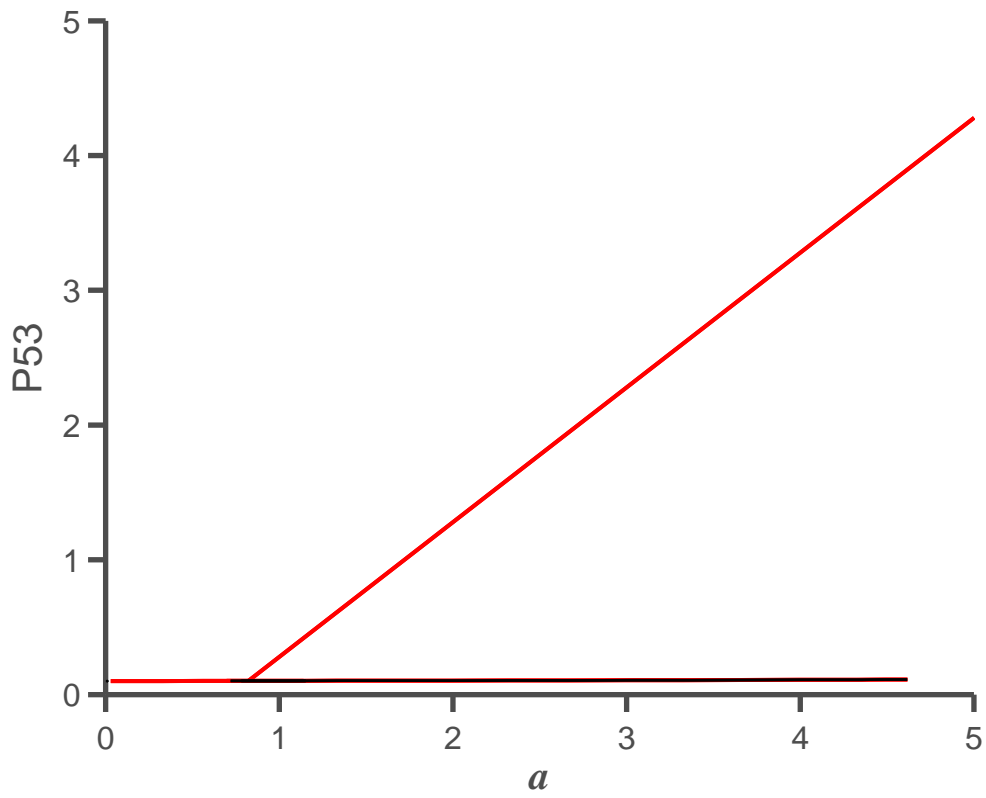

Supplement: S4 Fig — Red lines represent stable steady states. (PDF) [file pone.0197838.s004.pdf]

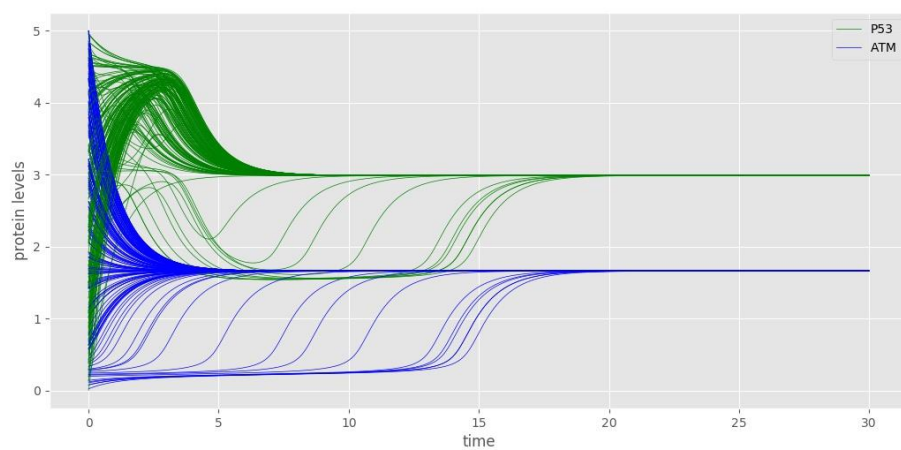

Supplement: S5 Fig — Time-course simulations for a = 1.5, b = 0.05, k = 1, S = 0.5 and n = 4 with random initial conditions which represent different cell states show that proteins converge to the stable steady state with a high p53 protein level indicating senescent state within about 20 hours. (PDF) [file pone.0197838.s005.pdf]

**S1 Table.**

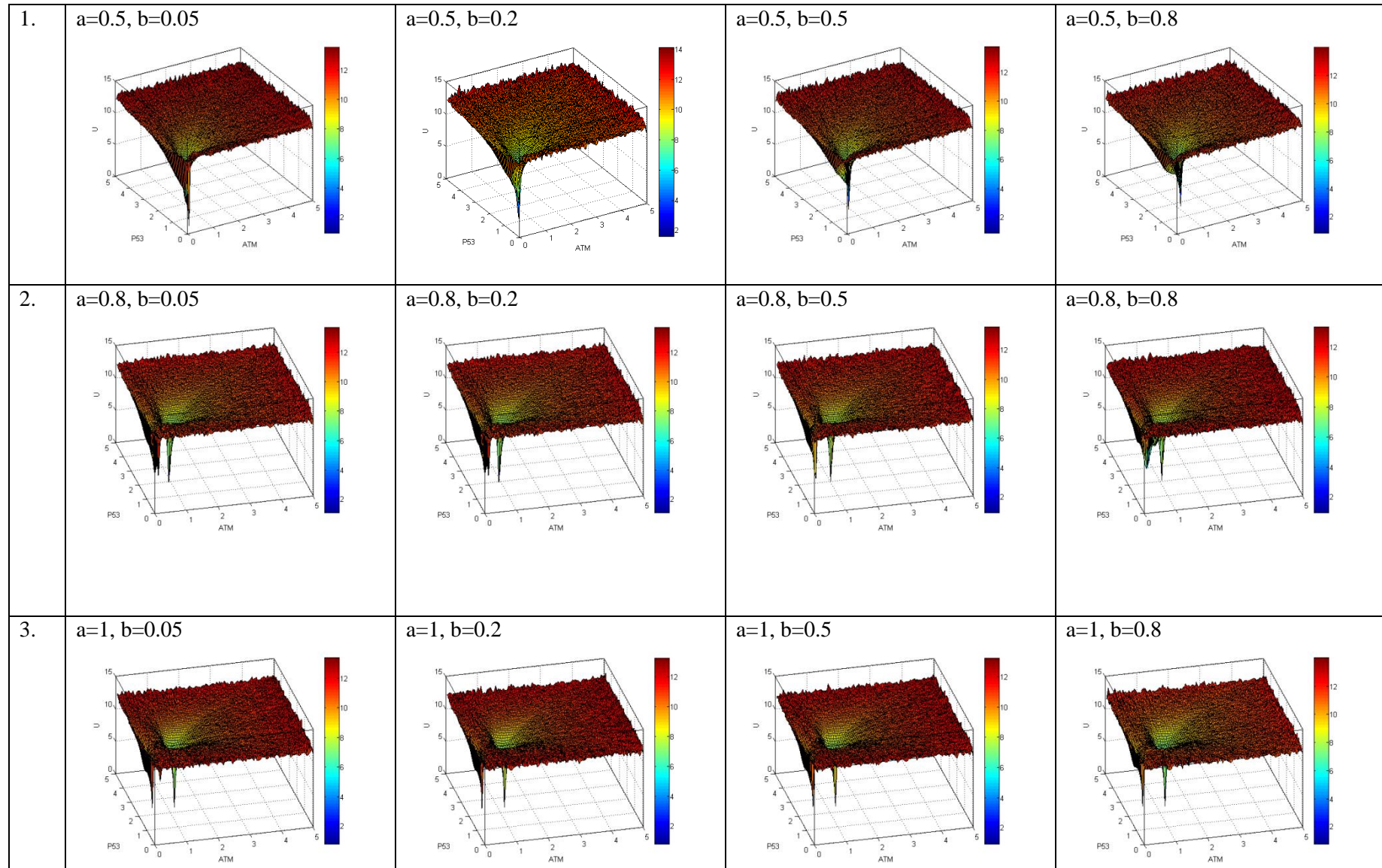

**S1 Table.**

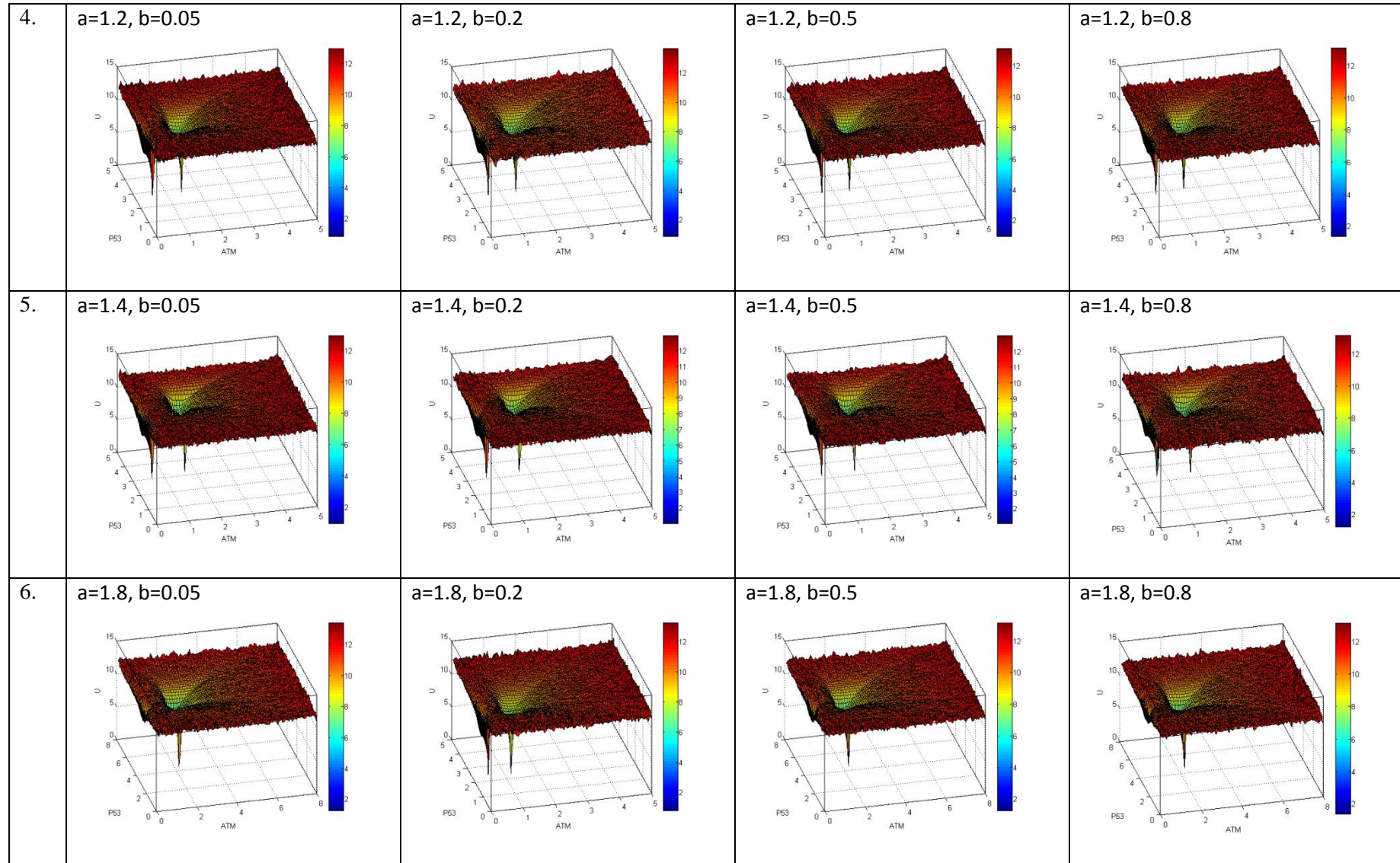

Supplement: S1 Table — Parameter sensitivity analyses were performed. For different combination of parameter values of a and b, the landscapes in 3-dimensional views are shown in the table. (PDF) [file pone.0197838.s006.pdf]

**S3 Table.**

|    |                                                                                                   |                                                                                                   |                                                                                                    |                                                                                                    |
|----|---------------------------------------------------------------------------------------------------|---------------------------------------------------------------------------------------------------|----------------------------------------------------------------------------------------------------|----------------------------------------------------------------------------------------------------|
| 1. | $a=0.5, b=0.05$ 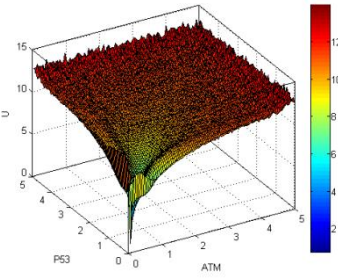 | $a=0.5, b=0.2$ 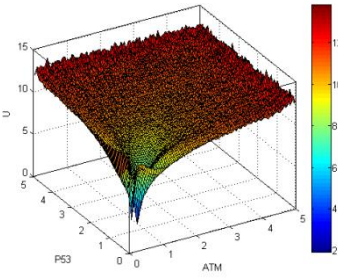 | $a=0.5, b=0.5$ 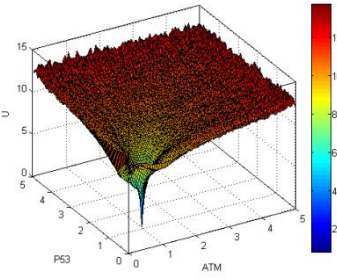 | $a=0.5, b=0.8$ 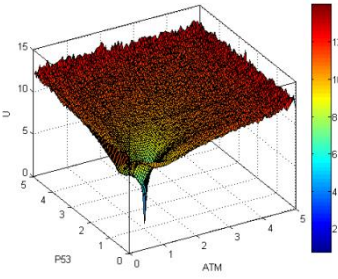 |
| 2. | $a=0.8, b=0.05$ 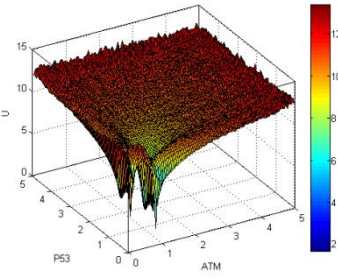 | $a=0.8, b=0.2$ 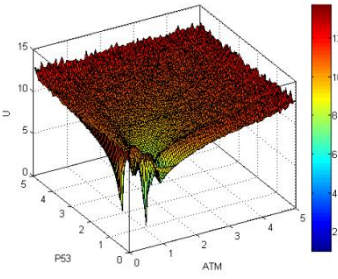 | $a=0.8, b=0.5$ 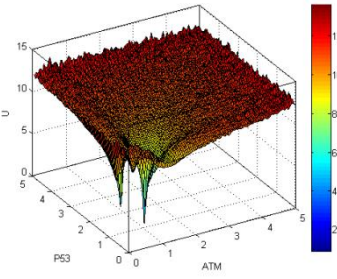 | $a=0.8, b=0.8$ 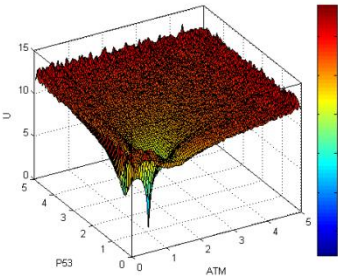 |
| 3. | $a=1, b=0.05$ 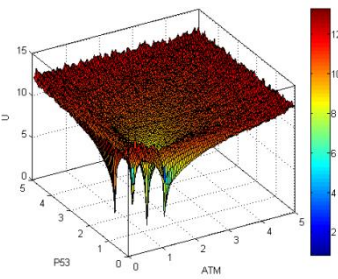 | $a=1, b=0.2$ 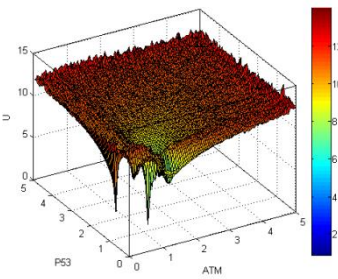 | $a=1, b=0.5$ 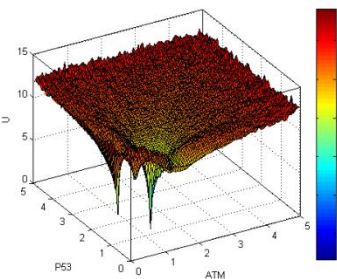 | $a=1, b=0.8$ 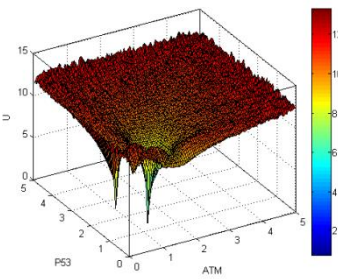 |

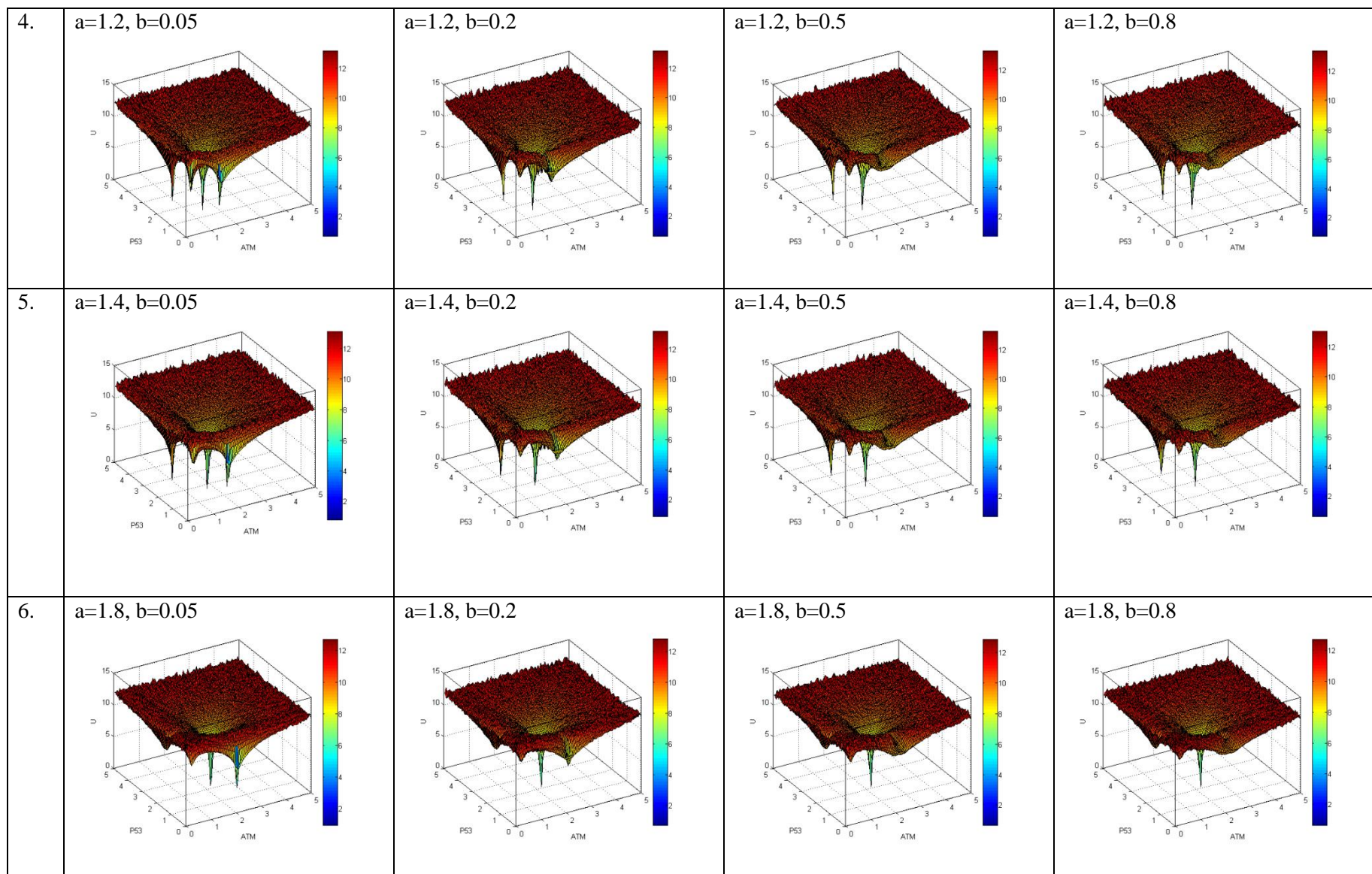

Supplement: S3 Table — Parameter sensitivity analyses were performed for the perturbed network. Potential landscapes in 3-dimensional views are shown in the table. (PDF) [file pone.0197838.s008.pdf]
