# Supplementary material for: Dynamical analysis of cellular ageing by modeling of gene regulatory network based attractor landscape
Source: PLoS One. 2018 Jun 1;13(6):e0197838. doi: 10.1371/journal.pone.0197838 (PMC5983441; doi:10.1371/journal.pone.0197838)
Supplement: S2 Table — In silico network perturbations were performed with a = 1.5 and b = 0.05. (PDF) [file pone.0197838.s007.pdf]

S2 Table.

No.	Perturbation	Top view	3D view U=- *ln* P(x)
1.	Deleted ATM-->p53 and ARF-->p53	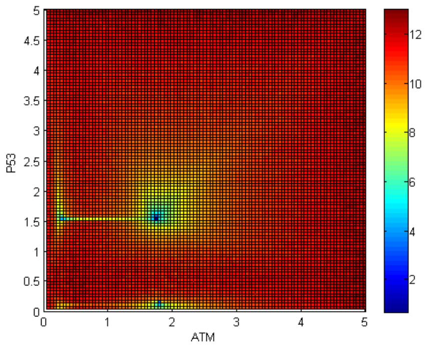	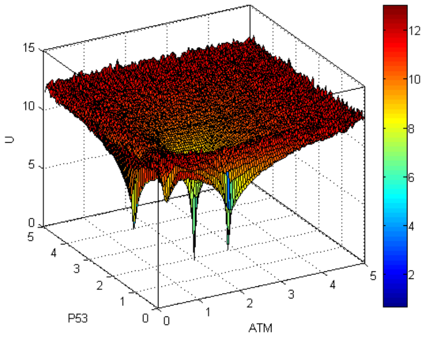
2.	Deleted ARF-->p53	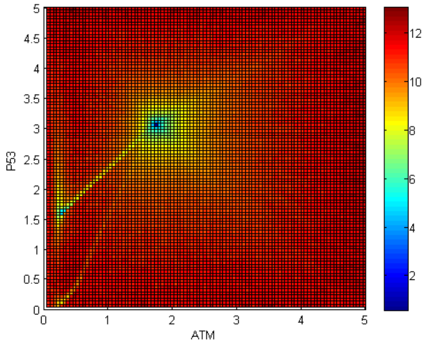	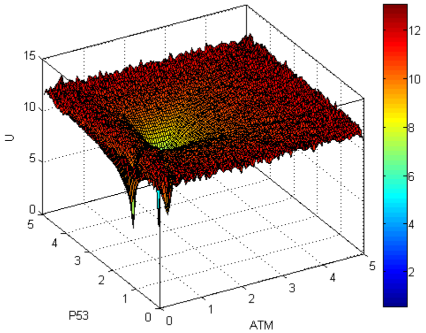

3.	Deleted p53-->p53	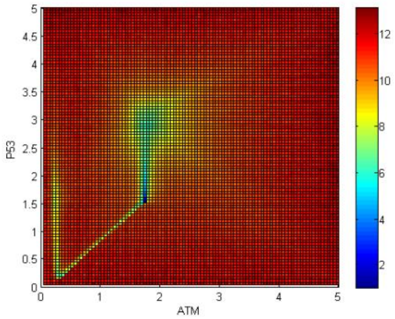	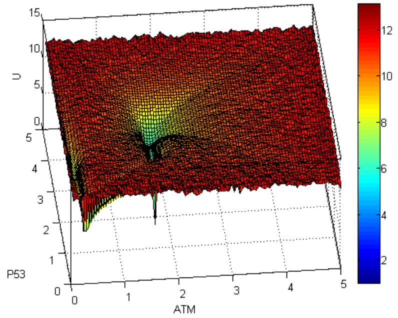
4.	Deleted ATM-->p53, ARF-->p53 and p53-->p53	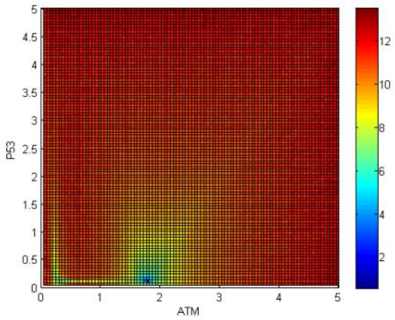	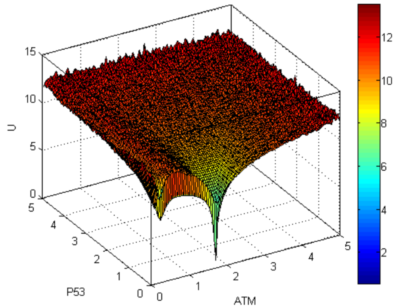
5.	Deleted ATM-->p53	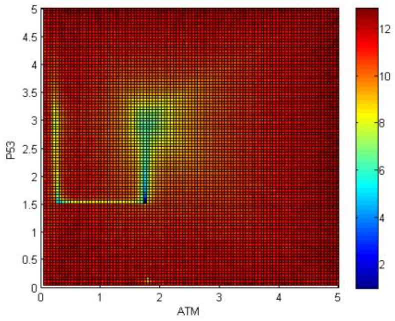	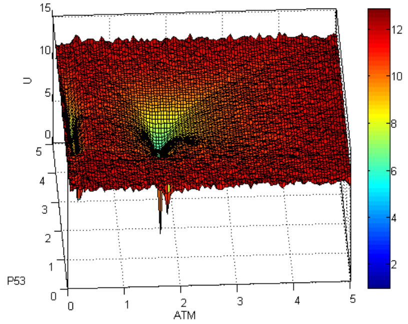
