# Supplementary material for: Dynamical analysis of cellular ageing by modeling of gene regulatory network based attractor landscape
Source: PLoS One. 2018 Jun 1;13(6):e0197838. doi: 10.1371/journal.pone.0197838 (PMC5983441; doi:10.1371/journal.pone.0197838)
Supplement: S1 Text — (PDF) [file pone.0197838.s009.pdf]

S1 Text

Dynamical Analysis of Cellular Ageing by Modeling of Gene Regulatory Network Based Attractor Landscape

Ket Hing Chong[1], Xiaomeng Zhang[1], Jie Zheng[1,2,*]

[1] Biomedical Informatics Lab, School of Computer Science and Engineering, Nanyang Technological University, 639798, Singapore
[2] Complexity Institute, Nanyang Technological University, 637723, Singapore
[*]zhengjie@ntu.edu.sg

We have tried using more realistic parameter values in our model. In particular, instead of using a common value of protein degradation rate *k* = 1, we used different values of *k* for different proteins, found in the literature (Table A). However, using the different values of *k*, we are unable to reproduce the expected multi-stability dynamics (i.e. of two stable attractors for cell cycle arrest and senescence) as shown in the landscape quantified with the common protein degradation rate *k* = 1.

The realistic protein degradation rates (in Table A) were estimated from the half-lives of the proteins as reported in the literature (for first-order reactions) using the following formula:

$$k = \frac{\ln(2)}{\mathrm{half-life}}$$

Table A. The estimated degradation rates for all the proteins in the ageing GRN.

No.	Protein	Half-life (hour)	Degradation rate *k* ($hour^{-1}$)	Protein Half-life Reference
1.	P53	4	0.173	[1]
2.	Mdm2	0.5	1.386	[2]
3.	Wip1	1.5	0.462	[3]
4.	ATM	4	0.173	[4]
5.	P21	0.5	1.386	[5]
6.	PTEN	7.5	0.092	[6]
7.	AKT	3	0.231	[7]
8.	Myc	0.5	1.386	[8]
9.	E2F	1.167	0.594	[9]
10.	RB	12	0.058	[10]
11.	CycE	0.5	1.386	[11]
12.	CycD	0.5	1.386	[12]
13.	ARF	0.5	1.386	[13]

The new landscapes, which were quantified and plotted using different values of *k* in Table A, each have only one attractor (Figure A and Figure B). Their shapes are totally different from the landscapes plotted using the common value *k* = 1, which show multi-stability (e.g. Figs 6B and 8). The landscape in Fig. 6A also has one attractor only.

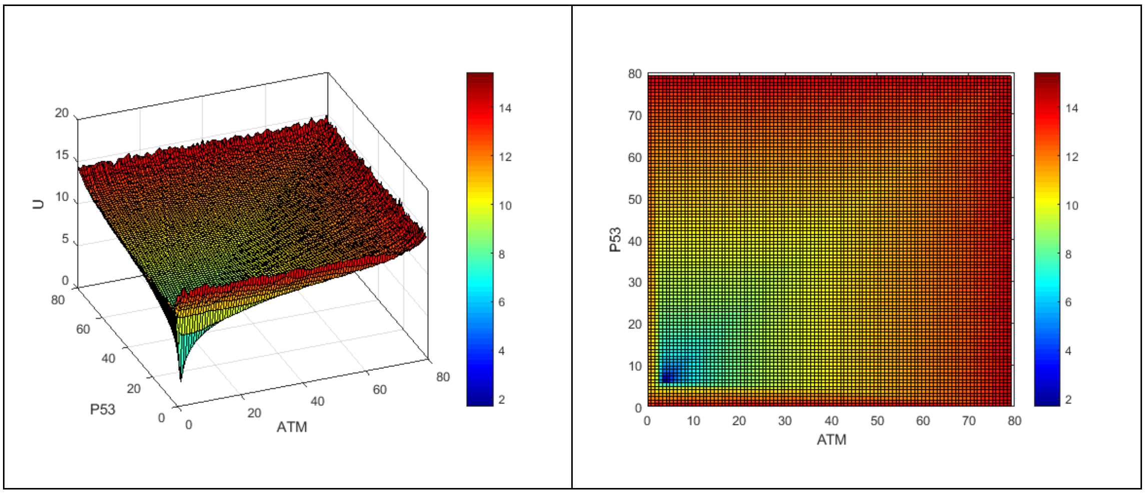

Figure A. Parameter values we chose here are: *S* = 0.5, *n* = 4, *k* equal to the values in Table A, *a* = 0.5 and *b* = 0.05.

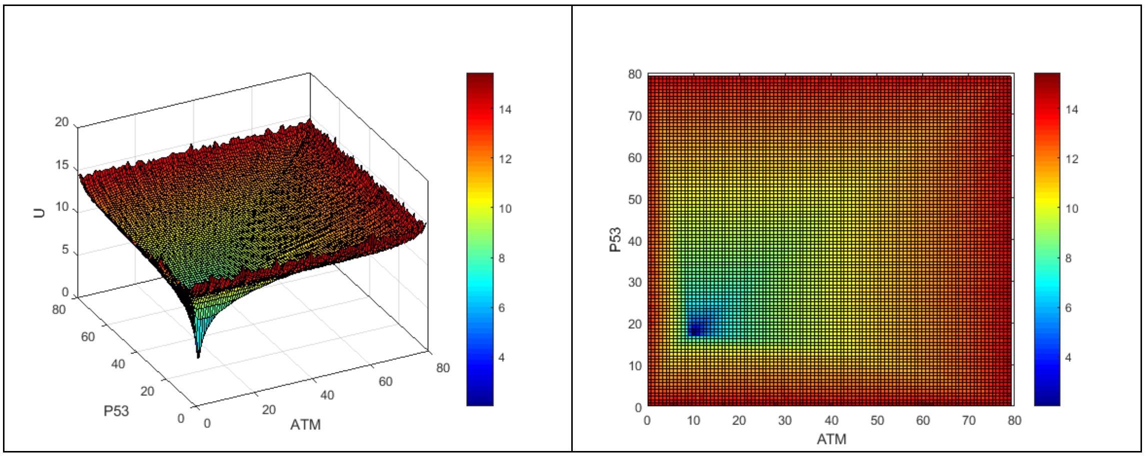

Figure B. Parameter values we chose here are: *S* = 0.5, *n* = 4, *k* equal to the values in Table A, *a* = 1.5 and *b* = 0.05.

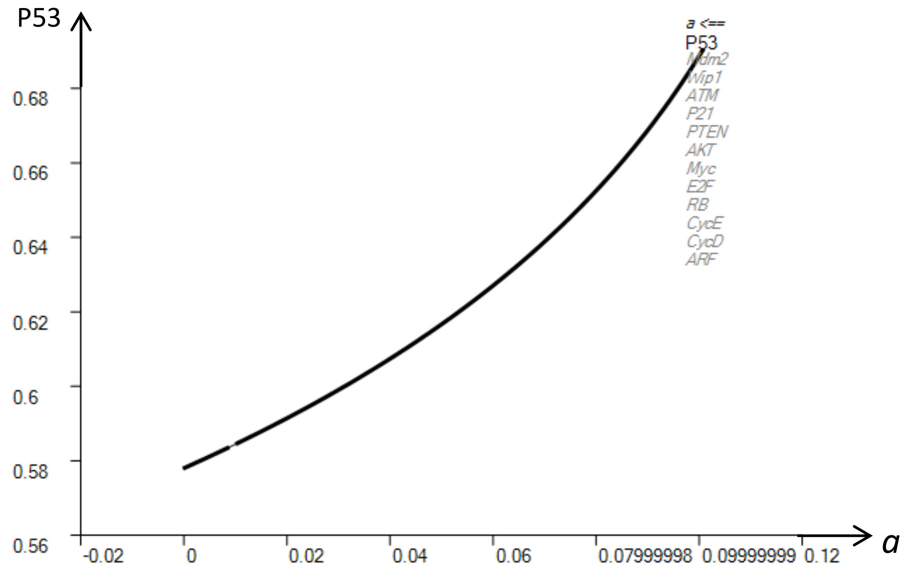

Figure C. Bifurcation diagram for p53 versus the parameter *a*. It shows that there is only one stable steady state when parameter *a* is increased.

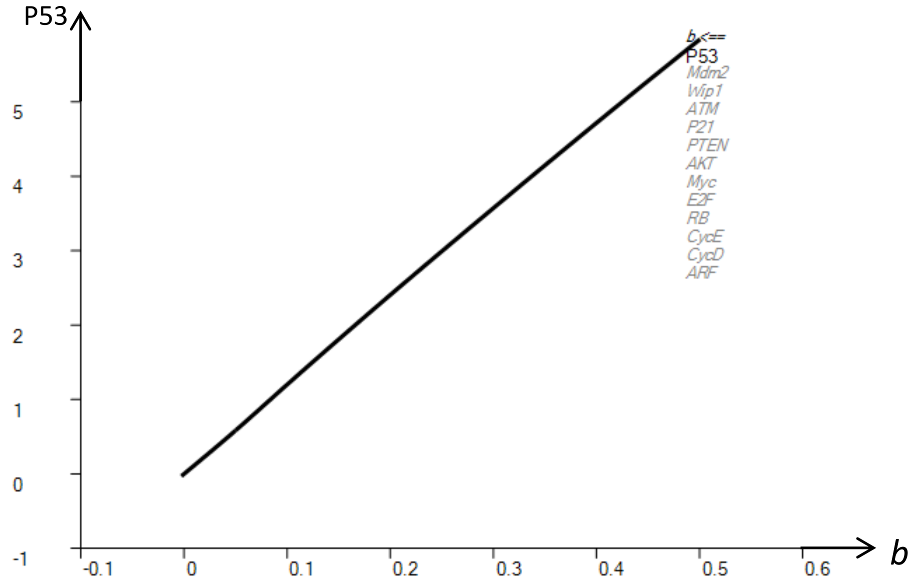

Figure D. Bifurcation diagram for p53 versus the parameter *b*. It shows that there is only one stable steady state when parameter *b* is increased.

The bifurcation diagrams for p53 versus the parameters *a* and *b* (Figure C and Figure D) indicate that the dynamical system only displays one stable steady state. Thus, it is not possible to get multi-stability with different values of protein degradation rates as given in Table A. With these results from the potential landscapes and bifurcation analyses we conclude that the model of ageing is not robust to the changes in values of the protein degradation rates.

In the main text, the value of $k = 1$ was adapted from a model constructed by Li and Wang [14]. Li and Wang referred to a model also using $k = 1$, previously proposed by Huang et al. (2007) [15].

References

1. Hubbert NL, Sedman SA, Schiller JT. Human papillomavirus type 16 E6 increases the degradation rate of p53 in human keratinocytes. J Virol. 1992;66(10):6237-41.
2. Peng Y, Chen L, Li C, Lu W, Agrawal S, Chen J. Stabilization of the MDM2 oncoprotein by mutant p53. J Biol Chem. 2001;276(9):6874-8.
3. Kleiblova P, Shaltiel IA, Benada J, Pecháčková S, Pohlreich P, Voest EE, et al. Gain-of-function mutations of PPM1D/Wip1 impair the p53-dependent G1 checkpoint. J Cell Biol. 2013;201(4):511-21.
4. Takai H, Wang RC, Takai KK, Yang H, de Lange T. Tel2 regulates the stability of PI3K-related protein kinases. Cell. 2007;131(7):1248-59.
5. Chang L-J, Eastman A. Decreased translation of p21waf1 mRNA causes attenuated p53 signaling in some p53 wild-type tumors. Cell cycle. 2012;11(9):1818-26.
6. Tamguney T, Stokoe D. New insights into PTEN. J Cell Sci. 2007;120(23):4071-9.
7. Fujio Y, Guo K, Mano T, Mitsuuchi Y, Testa JR, Walsh K. Cell cycle withdrawal promotes myogenic induction of Akt, a positive modulator of myocyte survival. Mol Cell Biol. 1999;19(7):5073-82.
8. Gregory MA, Hann SR. c-Myc proteolysis by the ubiquitin-proteasome pathway: stabilization of c-Myc in Burkitt's lymphoma cells. Mol Cell Biol. 2000;20(7):2423-35.
9. Campanero MR, Flemington EK. Regulation of E2F through ubiquitin–proteasome-dependent degradation: stabilization by the pRB tumor suppressor protein. Proceedings of the National Academy of Sciences. 1997;94(6):2221-6.
10. Darnell GA, Antalis TM, Johnstone RW, Stringer BW, Ogbourne SM, Harrich D, et al. Inhibition of retinoblastoma protein degradation by interaction with the serpin plasminogen activator inhibitor 2 via a novel consensus motif. Mol Cell Biol. 2003;23(18):6520-32.
11. Singer JD, Gurian-West M, Clurman B, Roberts JM. Cullin-3 targets cyclin E for ubiquitination and controls S phase in mammalian cells. Genes Dev. 1999;13(18):2375-87.
12. Kanie T, Onoyama I, Matsumoto A, Yamada M, Nakatsumi H, Tateishi Y, et al. Genetic reevaluation of the role of F-box proteins in cyclin D1 degradation. Mol Cell Biol. 2011:MCB. 06570-11.
13. Chen D, Shan J, Zhu W-G, Qin J, Gu W. Transcription-independent ARF regulation in oncogenic stress-mediated p53 responses. Nature. 2010;464(7288):624.
14. Li C, Wang J. Quantifying the Landscape for Development and Cancer from a Core Cancer Stem Cell Circuit. Cancer Res. 2015;75(13):2607-2618.
15. Huang S, Guo Y-P, May G, Enver T. Bifurcation dynamics in lineage-commitment in bipotent progenitor cells. Dev Biol. 2007;305(2):695-713. doi: http://dx.doi.org/10.1016/j.ydbio.2007.02.036.
